# Early results of a 6-week exercise programme in post-ICU patients

**DOI:** 10.1186/cc12479

**Published:** 2013-03-19

**Authors:** C Battle, K James, P Temblett, H Hutchings

**Affiliations:** 1 Morriston Hospital, ABMU Health Board, Swansea, UK; 2 Swansea University, Swansea, UK

## Introduction

The aim of this study was to investigate the effect of a 6-week exercise programme on outcomes in post-ICU patients. With improvements in intensive care medicine, increasing numbers of patients are surviving catastrophic illness [1]. Severe weakness is common in patients with prolonged critical illness and results in considerable morbidity, mortality and healthcare costs [2]. The NICE83 guidelines Rehabilitation in Critical Care recommend follow-up for post-ICU patients and that further research is needed in this field [3].

## Methods

Patients who have been discharged home from hospital following an ICU stay of 48 hours or more were recruited to the study. Patients were only excluded if they were not considered safe for exercise. Baseline measurements were completed prior to stratified (age, gender, APACHE II score) random allocation to either the exercise or control group. Outcome measures included cardiopulmonary fitness (6-minute walk test), balance (Berg Balance Scale), grip strength (JAMAR grip dynamometer) and hospital anxiety and depression (HAD score). The exercise group completed a 6-week supervised exercise programme, twice a week for up to 1 hour. In the seventh week, all patients repeated the baseline measurements. An unpaired Student's *t *test was used to compare any differences between the control and exercise groups.

## Results

At baseline measurements, there were no statistical differences in age, gender, length of stays or APACHE II scores between the two groups. Results indicate that the exercise group (*n *= 10) had significantly greater improvements in cardiopulmonary fitness (*P *0.001) and balance (*P *0.05) compared with the control group (*n *= 10). Greater improvements were also evident in anxiety, depression and grip strength in the exercise group, although not statistically significant.

## Conclusion

This pilot study highlights that a 6-week supervised exercise programme can significantly improve cardiopulmonary fitness and balance in post-ICU patients. Further recruitment to the study and 6-month/1-year follow-up is needed.
